# Humoral and Cell-Mediated Immune Response in Colostrum from Women Diagnosed Positive for SARS-CoV-2

**DOI:** 10.1089/bfm.2021.0082

**Published:** 2021-12-07

**Authors:** Vignesh Narayanaswamy, Brian Pentecost, Dominique Alfandari, Emily Chin, Kathleen Minor, Alyssa Kastrinakis, Tanya Lieberman, Kathleen F. Arcaro, Heidi Leftwich

**Affiliations:** ^1^ Department of Veterinary and Animal Sciences, University of Massachusetts, Amherst, Massachusetts, USA.; ^2^ Division of Maternal-Fetal Medicine, University of Massachusetts Medical School, Worcester, Massachusetts, USA.

**Keywords:** COVID-19, SARS-CoV-2, breastfeeding, colostrum, breast milk, antibody, cytokine

## Abstract

***Objective:*** To evaluate the immune response to severe acute respiratory syndrome coronavirus 2 (SARS-CoV-2) in colostrum from women who tested positive for the virus.

***Methods:*** Between March and September 2020 we obtained bilateral colostrum samples collected on spot cards within 48 hours of delivery from 15 new mothers who had previously tested positive for SARS-CoV-2. Four of 15 women provided liquid colostrum, which was used for validating results obtained from spot cards. Archived bilateral colostrum samples collected from 8 women during 2011–2013 were used as pre-coronavirus disease 2019 (COVID-19) controls. All samples were tested for reactivity to the receptor binding domain (RBD) of the SARS-CoV-2 spike protein using an enzyme-linked immunosorbent assay that measures SARS-CoV-2 RBD-specific IgA, IgG, and IgM and for levels of 10 inflammatory cytokines (interferon-gamma [IFN-γ], tumor necrosis factor-alpha, interleukin [IL]-1β, IL-2, IL-4, IL-6, IL-8, IL-10, IL-12, IL-13) using a multiplex electrochemiluminescent sandwich assay.

***Results:*** Our validation studies indicate that the levels of SARS-CoV-2-specific antibodies and the associated cytokines measured in liquid colostrum are comparable to levels eluted from spot cards. Bilateral colostrum samples from 73%, 73%, and 33% of the 15 COVID-19 mothers exhibited IgA, IgG, and IgM reactivity to RBD, respectively. In addition, symptomatic COVID-19 mothers had statistically significant elevated levels of 4 of the 10 inflammatory markers (IFN-γ, IL-4, IL-6, and IL-12) compared to asymptomatic COVID-19 mothers.

***Conclusions:*** A strong humoral immune response is present in the colostrum of women who were infected with SARS-CoV-2 before delivering. The evolution and duration of the antibody response, as well as dynamics of the cytokine response, remain to be determined. Our results also indicate that future large-scale studies can be conducted with milk easily collected on paper spot cards.

## Introduction

The Center for Disease Control and Prevention and the World Health Organization (WHO) recommend breastfeeding for mothers infected with severe acute respiratory syndrome coronavirus 2 (SARS-CoV-2), as the benefits of mother's milk are thought to outweigh potential risks of transmitting the virus to the infant.^1,2^ A recent systematic review reporting on 77 nursing mothers from 37 studies concluded that there was no convincing evidence of transmission of live SARS-CoV-2 through breast milk.^3^ As of May 28th, 2021, the WHO reported over 168 million people infected by SARS-CoV-2 globally and over 3.5 million deaths. As the number of pregnant and lactating SARS-CoV-2-infected women increases, there is a need to build on existing, yet limited, research on SARS-CoV-2-specific immune response in breast milk from infected women. Antibodies to SARS-CoV-2 and the associated cytokines in breast milk are relevant to the health of nursed babies and mothers.^1,2,4,5^ Previous studies have shown the presence of SARS-CoV-2-specific antibodies in transitional-to-mature milk obtained from women exposed to the virus.^4–7^ Thus far, only two studies determined virus-specific IgA in colostrum obtained from 14 women who tested positive for SARS-CoV-2.^8,9^ Colostrum is important to promote the offspring's immune function. The number of infants ever breastfed is ∼1.4 times higher than infants breastfeeding at 6 months.^10–12^ The protective qualities of colostrum conferred by constituent antibodies and cytokines have clinical implications regarding the discussion about breastfeeding after infection. For this reason, knowledge regarding SARS-CoV-2-associated immune factors in colostrum in addition to mature milk is warranted.

Multiple studies have reported an increase in inflammatory cytokines in the serum and bronchoalveolar lavage fluid of coronavirus disease 2019 (COVID-19)-infected individuals.^13–19^ However, with the exception of one case study,^20^ there are no other reports on the cytokine profiles in breast milk or colostrum of women diagnosed positive for SARS-CoV-2. We^21,22^ and others^23–26^ have previously measured cytokines in breast milk and colostrum. Published literature suggests the transfer of cytokines through breast milk, and colostrum can compensate for the delay in development of the infant immune system, conferring protection against various infectious diseases and allergies.^23–26^ Furthermore, prior studies suggest that ingestion of breast milk confers long-lasting protection to the infant to specific viral respiratory tract infections.^27–29^ The present study details findings regarding SARS-CoV-2-specific IgA, IgG, and IgM and cytokine profiles in bilateral samples of colostrum collected within the first 2 days after parturition from 15 women who had tested positive for SARS-CoV-2.

## Materials and Methods

### Recruitment of COVID-19-positive participants and selection of pre-COVID-19 controls

COVID-19-positive study participants were patients at UMass Memorial Medical Center (UMMC, Worcester, MA) and provided consent in accordance with an IRB-approved protocol (#H00020140). Fifteen participants who tested positive (COVID-19^pos^; by diagnostic quantitative reverse transcription polymerase chain reaction [RT-PCR]) provided bilateral colostrum on the day of or the day after delivery. The participants hand expressed colostrum from each breast onto spot cards (Whatman^®^ FTA^®^ card, Millipore Sigma, #WHAWB120205), which were left to dry at room temperature (RT).

We identified archived samples from eight women who donated liquid bilateral colostrum (1–3 days postpartum) during June 2011–May 2013. These samples were collected as part of another study aimed at determining cell types in milk collected at various stages of lactation. These colostrum samples were obtained following IRB-approved protocol (#1097).

### Processing bilateral colostrum samples on spot cards

Discs (6 mm diameter) prepared from spot cards were heat treated for 30 minutes at 56°C to inactivate any virus and were then resuspended in 500 μL of Tris-buffered saline with 0.1% Tween 20^®^ (TBST) in a 24-well plate. The plate was incubated with gentle shaking overnight at 4°C after which the TBST eluates were used for detection of anti-SARS-CoV-2-specific immunoglobulins. Extra eluates were stored at 4°C and used for the analysis of cytokines within 72 hours.

### Processing liquid bilateral colostrum from pre-COVID-19 samples to obtain a whey fraction

Briefly, 500 μL of colostrum was centrifuged at 820 *g* for 8 minutes. The whey fraction was transferred to a 2 mL centrifuge tube and heat treated for 30 minutes at 56°C. Samples from the whey fraction were used for the detection of anti-SARS-CoV-2-specific immunoglobulins.

### Enzyme-linked immunosorbent assay for the detection of anti-SARS-CoV-2 IgA, IgG, and IgM

A SARS-CoV-2 enzyme-linked immunosorbent assay (ELISA) was developed and validated at UMass Amherst. The receptor binding domain (RBD) spike protein cloned into the pCAGGS expression vector was expressed in HEK293T cells (ATCC) using PEI (10:1 PEI:DNA ratio) and purified by gravity flow, as described in Stadlbauer et al.^30^ Briefly, 96-well plates (#351172; Fisher Scientific) were coated with the RBD spike protein at 1 μg/mL in 1 × phosphate-buffered saline and incubated with gentle shaking overnight at 4°C followed by blocking in 5% (w/v) dry skimmed milk in TBST with gentle shaking for 30 minutes at RT. Fifty microliters of sample were added and incubated with gentle shaking for 1 hour at RT. Wells were then washed with TBST and incubated with horseradish peroxidase (HRP)-conjugated goat anti-human-IgA, goat anti-human-IgG, or goat anti-human-IgM at 1 μg/mL (Jackson Laboratory). Plates were washed thrice, incubated with 2,2′-Azinobis [3-ethylbenzothiazoline-6-sulfonic acid]-diammonium salt (ATBS, #A9941; Sigma Aldrich), diluted at 0.2 mg/mL in 0.1 M Sodium Acetate pH 4.5 at 37°C for 30 minutes. Known concentrations of anti-Spike-RBD-human (h) IgG1, -hIgM, and -hIgA1 (#C3022; InvivoGen, San Diego, CA) were assayed in the ELISAs. The concentration of the highest standard was 1,250 ng/mL; subsequent standards were prepared by 10-fold serial dilutions starting from 500 to 0.05 ng/mL. After background subtraction, concentration curves for IgA, IgG, and IgM were generated using a four-parametric logistic (4PL) curve with Excel's Solver Add-In. Concentrations of unknown samples were calculated using the 4PL equation.

### Analysis of cytokines

Cytokines were measured in a multiplex assay (Mesoscale Discovery, Gaithersburg, MD) according to the manufacturer's instructions using 10-plex human V-PLEX Proinflammatory Panel 1 plates. MSD assays use multispot plates allowing the quantitation of multiple targets in a single well. Each 96-well plate included an 8-point standard curve and assays for 10 cytokines: interleukin (IL)-2, IL-4, IL-6, IL-8, IL-10, IL-12p70, IL-13, IL-1β, interferon-gamma (IFN-γ), and tumor necrosis factor-alpha (TNF-α). Samples and standards were run in technical duplicates.

### Spotcards as a collection method to compare findings from liquid colostrum

Colostrum from four SARS-CoV-2 positive women was used to determine levels of anti-RBD antibodies and the associated cytokines in both the colostrum samples and laboratory-prepared spot card eluants. For simulating samples on spot cards, 125 μL of colostrum was pipetted onto each sample loading area (25.4 mm diameter), and the cards were left to dry overnight at RT. Discs (6 mm) were prepared and processed as described above. Based on the area of the disc we estimate that eluates are equivalent to 50 μL of a 1:20 dilution of the liquid colostrum sample. Therefore, to validate findings from spot cards, 1:20 dilutions in TBST of the four colostrum samples were prepared. Both the eluates and the diluted colostrum samples were assayed for anti-SARS-CoV-2 antibodies and the associated cytokines as described above.

### Data analysis

Welch's *t*-test was used to assess differences in age and body mass index (BMI) between donations made during 2020 and during 2011–2013. Wilcoxon single-rank test was used to compare analyte levels between spot card eluants and liquid colostrum. Colostrum was defined as positive for SARS-CoV-2-specific IgA, IgG, or IgM if the OD values for that assay were twice the mean OD levels for secondary-only antibody reactivities (background). Antibody titers were determined from 4PL curves generated for IgA, IgG, and IgM. Welch's *t*-test was used to compute differences in cytokines between symptomatic and asymptomatic participants. *p*-Values <0.05 were considered statistically significant after Bonferroni correction.

## Results

### Participant demographics

Demographic characteristics of the 23 women are summarized in Table 1, stratified by the period of colostrum donation: “2020” (COVID-19) versus “2011–2013” (Pre-COVID-19 controls). Groups did not differ significantly by age or BMI. Women who provided colostrum in 2020 self-identified as 31% Hispanic, 13% White Hispanic, 50% non-Hispanic White, and one woman identified as Asian American. The available prepandemic colostrum sample set, collected during 2011–2013, was exclusively from women who identified as White (one woman did not provide information on race).

**Table 1.** Demographics of Participants Who Donated Colostrum

	2020 (*n* = 15)		2011–2013 (*n* = 8*^a^*)		*p*
	Mean	Range	Mean	Range	
Age (year)	31	21–39	33	29–40	0.64
BMI (kg/m^2^)	34	22–42	27	22–33	0.08
	*n*	Percent	*n*	Percent	
Parity					
1	4	27	2	25	
2	5	33	4	50	
3	3	20	0	0	
4	—	—	1	12.5	
5	1	7	—	—	
Missing	2	13	1	12.5	
Race					
Asian American	1	6	0	0	
Hispanic	5	31	0	0	
White Hispanic	2	13	0	0	
White	8	50	7	88	
Missing	0	0	1	12	

^a^ BMI data not provided by 3 out of 8 participants.

BMI, body mass index.

**FIG. 1.**
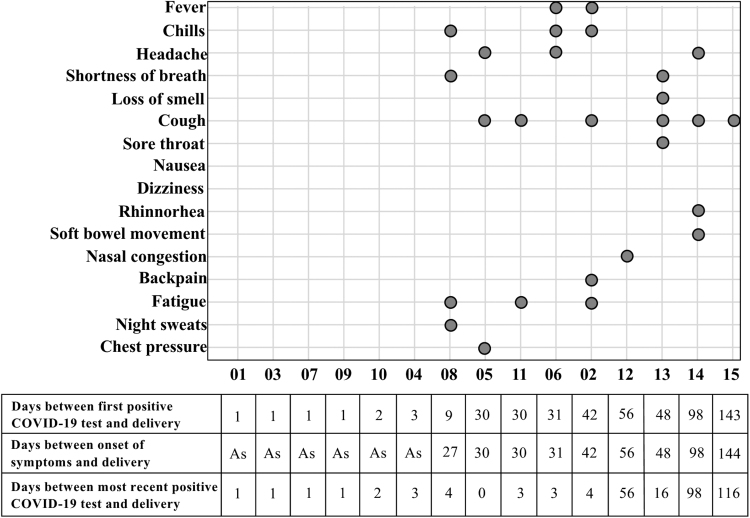
Overview of participants' COVID-19 symptoms relative to their time of delivery. Participants 01, 03, 04, 07, 09, and 10 reported no COVID-19-related symptoms (indicated as As—Asymptomatic) despite positive PCR tests. COVID-19, coronavirus disease 2019. PCR, polymerase chain reaction.

Eleven of the 15 participants tested positive for COVID-19 near the time of delivery [0–4 days (Fig. 1)]. The four participants who did not test positive near the time of delivery (12, 13, 14, and 15) had their most recent positive test 16 to 116 days before delivery (Fig. 1). Six of the 15 COVID-19^pos^ participants were asymptomatic. All of the participants reporting no symptoms (01, 03, 04, 07, 09, and 10) had their first positive test within 1–3 days of delivery. Onset of symptoms for the remaining 9 participants occurred between 27 and 144 days before delivery (Fig. 1). Eight of these nine participants reported that onset of symptoms occurred at the time of the first positive test; 08 reported onset of symptoms 18 days before her first positive test.

### Levels of anti-RBD antibodies and associated cytokines are similar in spot card eluants and liquid samples

**FIG. 2.**
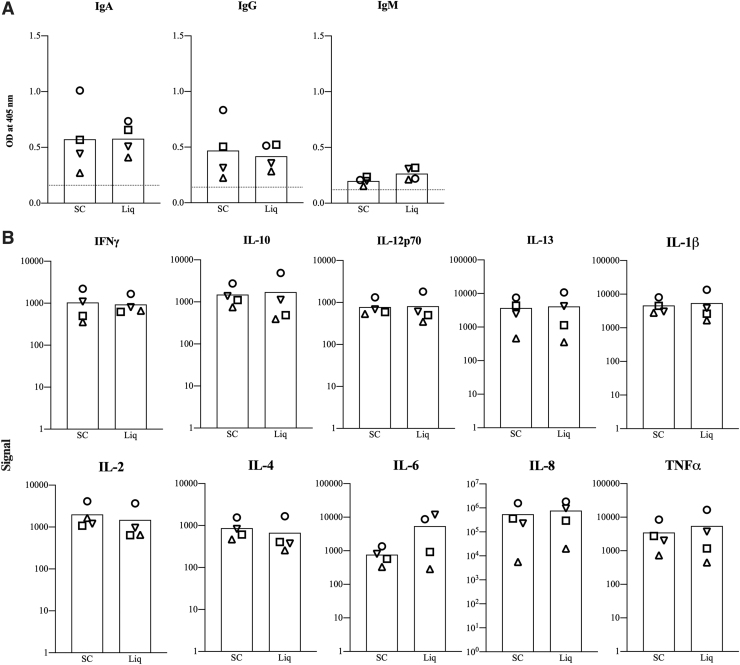
Similar levels of anti-RBD antibodies and associated cytokines in spot card eluants and corresponding liquid colostrum. Data were acquired for liquid and laboratory-prepared spot card eluants. Samples were assayed for anti-RBD IgA, IgG, and IgM **(A)** and for 10 cytokines **(B)**. Each bar indicates mean OD values obtained for spot card eluants (SC) or the equivalent liquid colostrum (Liq) provided by four women who had tested positive for SARS-CoV-2. Each symbol indicates a sample from a single woman. Wilcoxon single-rank test revealed no statistical difference in analyte levels between spot card eluants and liquid colostrum (*p* > 0.05). RBD, receptor binding domain; SARS-CoV-2, severe acute respiratory syndrome coronavirus 2.

Validation studies of antibodies and cytokines in spot cards utilized colostrum samples collected within 48 hours of delivery from four women who tested positive for SARS-CoV-2. Figure 2 shows that the binding reactivities of IgA, IgG, and IgM to RBD were similar in spot card eluants and liquid colostrum samples (*p* > 0.05) using equivalent amounts of samples. To validate levels of cytokines in spot cards, levels of 10 cytokines were measured in spot card eluants and in 1:20 diluted colostrum samples. Levels of all 10 cytokines were similar between spot card eluants and liquid colostrum samples (*p* > 0.05).

### Colostrum obtained from COVID-19^pos^ participants exhibited strong reactivity to anti-RBD IgA, IgG, and IgM

IgA, IgG, and IgM reactivities in bilateral colostrum samples on spot cards were measured in spot card eluants. Samples were collected within 48 hours of delivery from 15 women who had positive SARS-CoV-2 (COVID-19^pos^) tests. Eight liquid bilateral colostrum samples donated during 2011–2013 served as prepandemic controls. All samples were tested in technical replicates. The high precision of the assay is reflected in the low mean coefficient of variation (CVs): 3.0% for IgA, 2.9% for IgG, and 4.8% for IgM (Supplementary Fig. S1**)**.

**FIG. 3.**
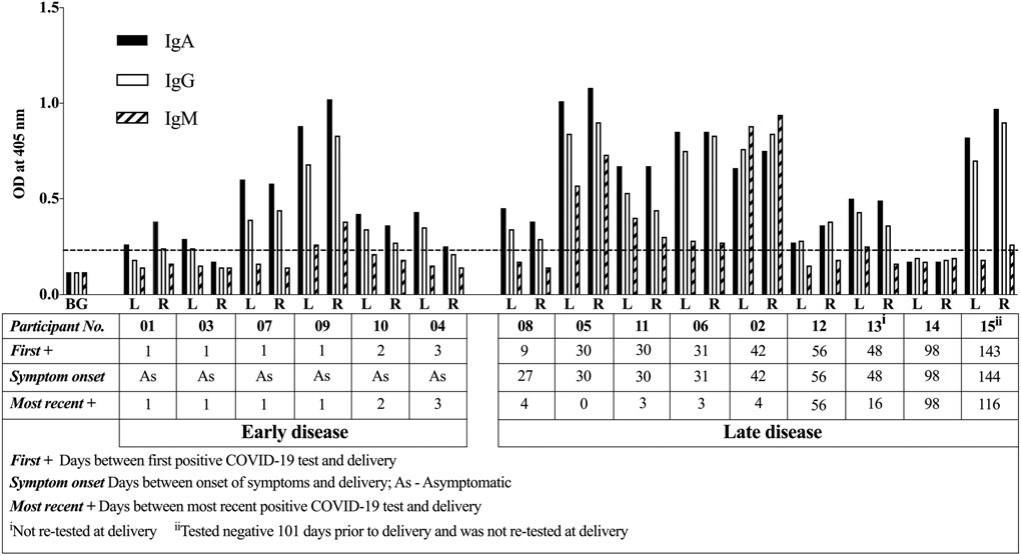
Distinct reactivities for IgA, IgG, and IgM in colostrum from COVID-19^pos^ participants. Bars indicate mean OD values for IgA, IgG, and IgM in colostrum from 15 participants who provided bilateral colostrum on spot cards. All spot card samples were collected within 48 hours postpartum. Participants tested negative, positive, or were not tested at delivery. Time between delivery and first positive diagnostic test, onset of symptoms, and most recent positive diagnostic test are presented. *Dotted lines* indicate cutoff value set at twice the mean OD of secondary-only antibody reactivity across all plates. OD values for all samples are the means of technical duplicates.

The binding reactivities of IgA, IgG, and IgM were similar for colostrum obtained from left and right breasts (Fig. 3). Figure 3 shows that colostrum was reactive to the RBD spike protein in samples from 14 of 15 participants for IgA and IgG and 6 of 15 for IgM. Only the colostrum from one COVID-19^pos^ participant (14) had no reactivity to the RBD spike protein.

Participants who were diagnosed positive by PCR test at delivery did not report experiencing any symptoms. Six participants (01, 03, 04, 07, 09, and 10; early disease group) had their first positive diagnostic test 1–3 days prior delivery, and none reported any symptoms, whereas eight out of nine participants (all but 08; late disease group) whose first diagnostic test was >25 days before delivery reported symptoms (Figs. 1 and 3). Among the participants who were symptomatic, all but 08 exhibited symptoms during the time of their first positive test, whereas 08 exhibited symptoms 18 days prior her first positive test. In contrast, neither the time since first positive test nor whether the participant experienced symptoms were related to the antibody reactivity to RBD spike protein. Among women who tested positive at the time of delivery, the highest reactivities occurred in participants 05 and 09, who had their first positive diagnostic viral PCR test 30 days (symptomatic) and 1 day (asymptomatic), respectively, before delivery (Fig. 3). Among the six women who did not have a positive test at delivery (02, 08, 12, and 14 tested negative at delivery; 13 and 15 were not retested at delivery), all of whom had symptomatic infection, the highest reactivity to RBD occurred in 02 and 15, who had their first positive diagnostic test 42 and 143 days, respectively, before delivery, while participant 14 had no antibody-reactivity and her first positive diagnostic test was 98 days before delivery (Fig. 3). The median IgA, IgG, and IgM concentrations were 67.11 versus 16.51 ng/mL; 38.76 ng/mL in spot card eluates of colostrum (Supplementary Fig. S2).

Bilateral colostrum from 2 of 8 pre-COVID-19 control participants (22 and 23) exhibited reactivities for IgA and IgG. Colostrum from the left breast of pre-COVID-19 control, 19, also exhibited reactivities for IgA and IgG, although low (Supplementary Fig. S3).

### Inflammatory markers in colostrum from symptomatic and asymptomatic COVID-19^pos^ participants

To further examine immune responses following SARS-CoV-2 infection, we measured cytokines in bilateral colostrum provided on spot card eluants. Analytes were generally present at detectable levels in samples; TNF-α, IL-2, IL-8, and IL-13 were detected in all samples (Table 2).

**Table 2.** Levels of Cytokines in Colostrum Provided on Spot Cards from 15 Severe Acute Respiratory Syndrome Coronavirus 2-Infected Women

Analytes	Mean signal (left breast)	Mean signal (right breast)	% Det^a^
IFN-γ	651	819	84.4
TNF-α	1769	2455	100
IL-1β	5127	10694	84.4
IL-2	1477	2254	100
IL-4	677	963	93.8
IL-6	463	795	84.4
IL-8	580014	755190	100
IL-10	1012	1225	96.7
IL-12	634	973	96.7
IL-13	1726	2396	100

^a^ percent detectable.

IFN-γ, interferon-gamma; IL, interleukin; TNF-α, tumor necrosis factor-alpha.

**FIG. 4.**
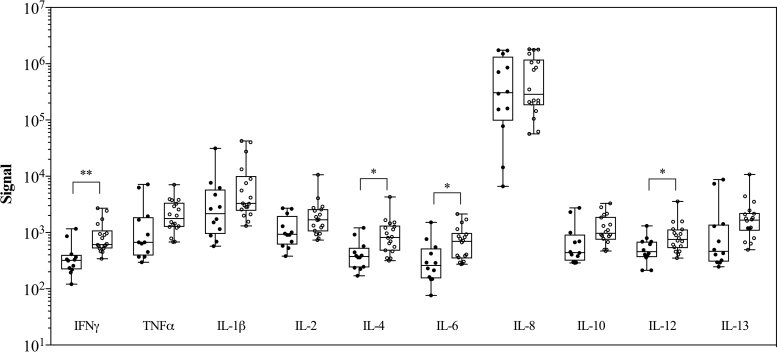
Higher levels of inflammatory cytokines in colostrum obtained from symptomatic COVID-19^pos^ participants. Comparison of levels of cytokines in spot card eluants from the 15 COVID-19^pos^ participants, stratified by whether participants experienced symptoms. The plots show the signal detected for each cytokine. *Open circles* indicate bilateral colostrum provided by participants who were symptomatic, and *Filled circles* indicate bilateral colostrum provided by participants who were asymptomatic. *p < 0.05, **p < 0.01.

We compared the cytokine levels between colostrum from symptomatic and asymptomatic participants. Symptomatic and asymptomatic women did not differ significantly with respect to age (*p* = 0.39) or BMI (*p* = 0.95) (data not shown). Symptomatic women had higher mean levels for 9 of the 10 analytes (all but IL-8) (Fig. 4). The levels of IFN-γ, IL-4, IL-6, and IL-12 were significantly higher in colostrum obtained from those participants who exhibited COVID-19-related symptoms (Fig. 4).

## Discussion

Our results provide a snapshot of the immune response in colostrum following SARS-CoV-2 infection, paralleling recent findings on the presence of SARS-CoV-2-specific antibodies in milk from infected women,^4–6,8^ and describing for the first time the associated cytokine profile. Colostrum samples archived before the pandemic together with analysis of bilateral samples provide important controls for this study, and the results from the spot card colostrum demonstrate the value of the collection method.

Spot card colostrum from all but one of the 15 COVID-19^pos^ participants exhibited IgA and IgG reactivities to RBD. The similarity in reactivity levels between the two breasts provides confidence in the assay; comparable levels of immunoglobulins across breasts are expected^31^ except when there are local infections. The extent of IgA, IgG, and IgM reactivities to RBD is not a direct function of the time since onset of symptoms. This is evidenced by the single COVID-19^pos^ participant who had onset of symptoms 98 days before delivery but whose colostrum had no antibody reactivity to RBD (14), while participant 15 with high levels of IgA and IgG reactivities to RBD had onset of symptoms 144 days before delivery (Fig. 3).

The immunoglobulin profile reported in colostrum is similar to that found in breast milk; ∼2% of colostrum antibody is IgG, ∼90% IgA, and 8% IgM. Nearly all colostrum IgA and IgM are in secretory form (sIgA and sIgM), conjugated to j-chain and secretory component (SC).^32^ Mammary tissue-resident plasma cells, which are sources of colostrum antibodies, are part of the gut-associated lymphoid tissue.^32,33^ The SC is essential for protecting sIgA and sIgM from the harsh environments of the infant stomach.^33^ Milk antibody composition and specificity are distinct from that found in blood. We did not compare antibody levels in the blood with colostrum; however, it is clear from our findings that colostrum from 14 out of 15 infected women contained SARS-CoV-2-reactive IgA without containing measurable levels of IgM, which is likely to be derived from the serum.

The presence of SARS-CoV-2-specific IgA and IgG in bilateral liquid colostrum of two pre-COVID-19 controls, and the left breast of a third control (participant 19), suggests a prior infection that elicited a humoral response that cross-reacted with SARS-CoV-2-RBD **(**Supplementary Fig. S1A). This would be consistent with findings from Pace et al., who demonstrated that levels of SARS-CoV-2-specific milk IgA and IgG correlated with IgA and IgG concentrations specific to the S-protein of 229E coronavirus.^4^ Similar findings were observed by Anderson et al., who determined that 20% of individuals who provided serum samples before the pandemic possessed non-neutralizing antibodies against SARS-CoV-2 spike and nucleocapsid proteins.^34^ Consistently, Isho et al. observed elevated saliva IgA and IgG reactivities to SARS-CoV-2-RBD among their pre-COVID-19 controls and speculate that these are cross-reactive antibodies.^35^ We were concerned that the reactivity in the left breast of P19 could have been due to experimental error; however, a repeat analysis confirmed the IgA and IgG reactivities.

Our findings indicate that the levels of IFN-γ, IL-4, IL-6, and IL-12 are significantly higher in colostrum from symptomatic SARS-CoV-2-infected women compared to asymptomatic women (Fig. 4). The interferon response acts as the first line of defense against viruses. Innate immune sensors recognize SARS-CoV-2 RNA, activating the transcription factors nuclear factor-κB (NF-κB) and interferon regulatory factors (IRFs). Activation of NF-κB and IRFs stimulate production of pro-inflammatory cytokines and interferons. Secreted interferons induce expression of interferon-stimulated genes using the JAK-STAT pathway.^36^ Published literature suggests that in severe COVID-19 cases, many viral proteins antagonize this interferon response pathway, while still eliciting pro-inflammatory cytokines.^13,37,38^ A meta-analysis carried out on 264 COVID-19 patients, of whom 123 had severe disease, found that the IL-6/IFN-γ ratio was found to be significantly increased in those patients with severe disease.^39^ On the contrary, Tanacan et al. reported that IFN-γ levels positively correlated with disease severity in pregnant women with COVID-19 and that significantly higher levels of IFN-γ were found in the third trimester.^40^ The nature and linkage to disease severity of changes in IFN-γ levels in colostrum of COVID-19 infected lactating women need further investigation with a larger sample size.

Spot cards provide an efficient means of collecting colostrum and are easy to process as they are left to dry at RT for several hours. We are the first to utilize spot cards to determine levels of antibodies and cytokines in the colostrum obtained from SARS-CoV-2-exposed women. Our validation studies indicate that colostrum applied to and eluted from FTA™ spot cards provide data comparable to that from liquid samples (Fig. 2). Use of FTA™ spot cards provides a facile option for serial collection of colostrum and milk in future studies for analysis of both nucleic acids and protein.

The possible protection of infants from SARS-CoV-2-specific antibodies detected in colostrum has clinical implications regarding the discussion about breastfeeding after exposure to the virus. SARS-CoV-2-specific immune response was detected in the colostrum of women who had their first positive test and symptoms more than 4 months before delivery, women who were symptomatic at delivery, as well as asymptomatic women who had a first positive test at delivery. The detection of SARS-CoV-2-specific antibodies in these women with diverse COVID-19 disease experiences provides objective data for the value of initiating breastfeeding despite SARS-CoV-2 infection.

### Limitations

First, the samples analyzed were collected at a single time point and provide a glimpse of the immune response in colostrum associated with SARS-CoV-2 infection at the time of sample collection. Long-term follow-up studies are necessary to understand the dynamic changes as a result of this infection. Second, we did not perform functional assays to determine the ability of SARS-CoV-2-specific antibodies to neutralize the virus.

## Conclusion

Our study is among the first to demonstrate the presence of SARS-CoV-2-specific antibodies in colostrum and describes for the first time the cytokine profile in colostrum from women infected with SARS-CoV-2. The evolution and duration of the antibody response, as well as dynamics of the cytokine response, remain to be determined. Given the feasibility of the collection method, and the ability to detect antibodies and cytokines, our results indicate that future large-scale studies can be conducted with milk easily collected on paper spot cards.

## Ethical Approval and Consent to Participate

Approved by IRBs at UMMC to HL (H00020140) and at UMass Amherst to K.A. (2075).

## Supplementary Material

Supplemental data
