# Supplementary material for: Humoral and Cell-Mediated Immune Response in Colostrum from Women Diagnosed Positive for SARS-CoV-2
Source: Breastfeed Med. 2021 Dec 7;16(12):987–94. doi: 10.1089/bfm.2021.0082 (PMC8713451; doi:10.1089/bfm.2021.0082)
Supplement: Supplemental data [file Supp_Fig1-3.docx]

**Humoral and cell-mediated immune response in colostrum from women diagnosed positive for SARS-CoV-2**

Vignesh Narayanaswamy *et al*

**Figure legends for supplemental figures**

**
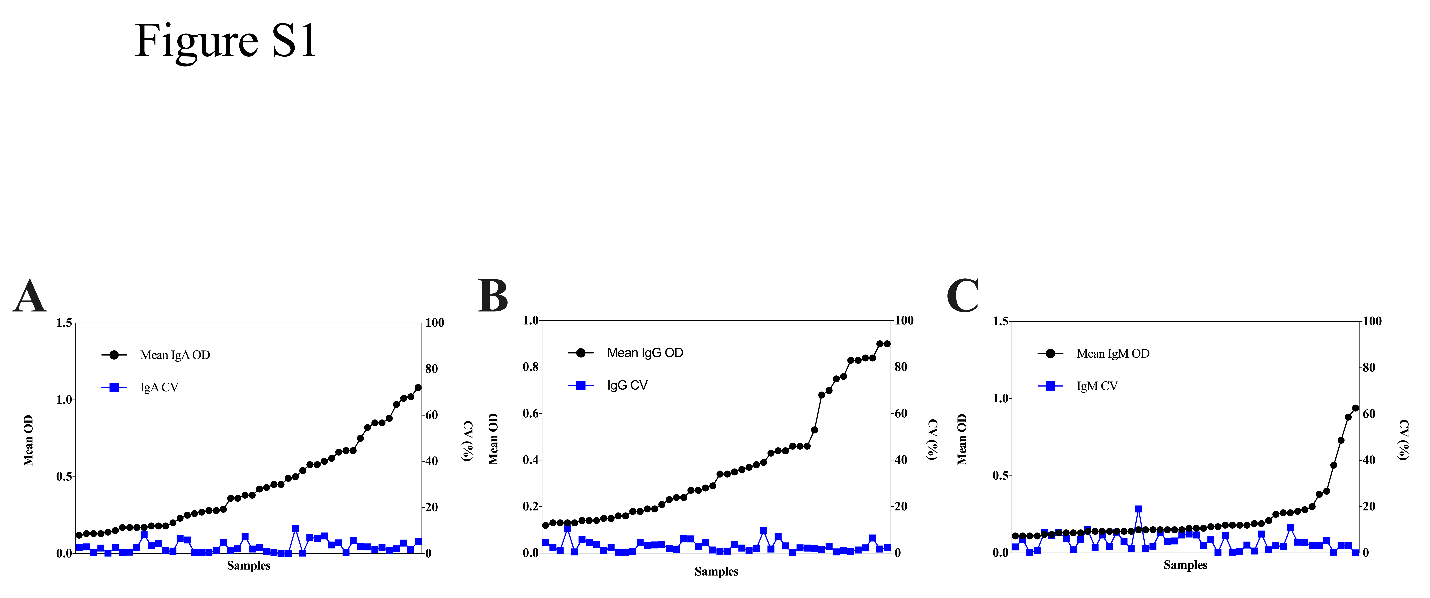
Figure S1. Performance evaluation of the SARS-CoV-2 specific IgA, IgG and IgM ELISA assay.** Reactivities to IgA (A), IgG (B) and IgM (B) (black circles) and their corresponding coefficient of variation (CV) (blue boxes) of the 46 colostrum samples (from the left and right breasts of 23 women). Each sample was tested in technical duplicate and the CV of duplicate OD values was calculated.

**
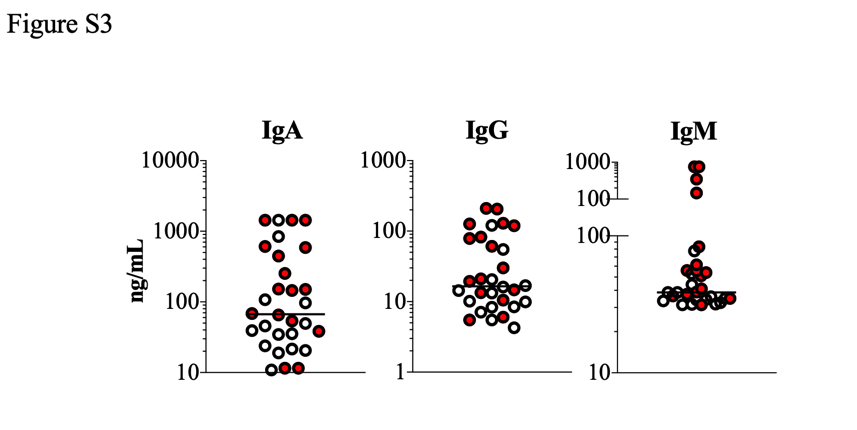
Figure S2. Levels of IgA, IgG and IgM in COVID-19 participants**. Concentrations of IgA (A), IgG (B) and IgM (C) were calculated from ODs obtained for spot card colostrum samples using a four-parametric logistic curve generated with Excel’s Solver Add-In (see Methods). Red circles indicate bilateral colostrum provided by participants who exhibited COVID-19-related symptoms.

**
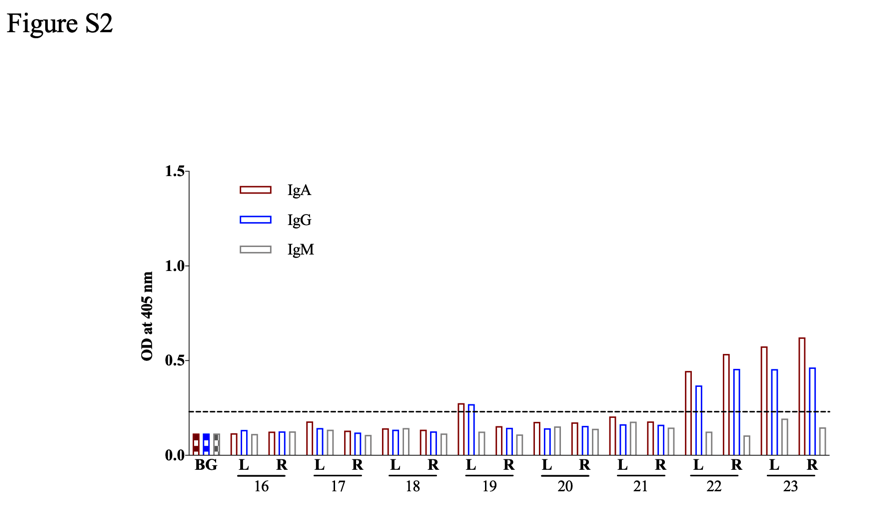
Figure S3.** **Reactivities for IgA, IgG and IgM in colostrum from pre-COVID-19 participants.** Mean OD values for IgA, IgG and IgM in bilateral liquid colostrum obtained from 8 pre-COVID-19 control participants. All liquid colostrum was collected 1-3 days postpartum.
